# Decision criteria for the selection of analytical instruments used in clinical chemistry: II Definition of problems, types of instruments and their selection

**DOI:** 10.1155/S1463924680000089

**Published:** 1980

**Authors:** Frederick L. Mitchell

**Affiliations:** Division of Clinical Chemistry Clinical Research Centre Harrow Middlesex HA1 3UJ UK


### Decision criteria for the selection of analytical instruments used in clinical chemistry

### II Definition of problems, types of instruments and their selection

**Frederick L. Mitchell**
*Division of Clinical Chemistry, Clinical Research Centre, Harrow, Middlesex HA1 3UJ, U.K.*

THE impact of the current technological revolution on clinical chemistry has been dramatic and continues to be so. Two decades of its influence have not only produced a bewildering array of purpose designed, complex and expensive instruments, but also a lucrative market both for vendors of instruments and systems, and for those who use them in the public or private sectors to provide laboratory health care facilities to patients.

For all concerned, it is important that the best possible instrumentation is available and is purchased in all circumstances.

### Definition of problem

A decision to purchase equipment must be made with the appreciation of a problem in the laboratory which might arise under one or more of the following headings:-

*Capacity*
Workload outstripping capacity.

*Replacement*
(i) Old instruments becoming unreliable or maintenance too costly
(ii) Obsolescent instruments using too much sample or reagent
(iii) Poor quality control performance indicating that better instruments are required.

*New technology*
New techniques require a new approach

*Reorganisation*
An old laboratory requires bringing up to date in instrumentation, computer control or data processing.

*Emergency work*
(i) Emergency specimens interfering with routine runs.
(ii) Emergency work inaccurate, imprecise or not completed sufficiently rapidly.

*Status symbol*
Equipment not sufficiently prestigious to attract adequate work in a commercial laboratory.

Whatever the problem, it is important that it should be carefully defined and the value of new instrumentation as a solution to it should be assessed. A clear and well-reasoned case is invariably required by the finance-providing authorities and is usually important for instrument selection.

### General considerations

It is only human nature for every laboratory head to consider his particular case unique. There is indeed some substance in this belief since, even if there are others with laboratories of the same size, situation, clientele, etc., he will have gathered around him staff of a character and aptitude which suit his own; in these respects we are all individuals. Additionally, in a developing science, any tendency to enforce regimentation must be resisted if development to the benefit of all is to continue in the best possible way. Therefore, choice must rest with the individual; the laboratory head must be provided with all the information he needs and be placed in the best possible position to make his choice.

### Categories

All users, instruments, systems and situations cannot be discussed under one heading, so that for the purposes of this discussion it is convenient to make divisions into the following categories.

A. Size of laboratory
  (1) Large : specimens received per day more than 300
  (2) Medium : specimens received per day, between 50 and 300
  (3) Small : specimens received per day, less than 50

B. Types of instruments available for chemical assay
  (1) Continuous flow
  (2) Discrete
  (3) Discretionary
  (4) Non-discretionary
  (5) Large multi-channel
  (6) Smaller multi-channel instruments (sometimes assembled by multiplexing single channel machines to suit the operator)
  (7) Single channel fast analysers operating in both continuous and batch mode
  (8) Single channel continuously operating relatively slow instruments
  (9) Instruments operating with completely pre-packaged reagents
  (10) Stand-alone automatic instruments for measuring one, or a small number of analytes, e.g. glucose, urea or electrolyte analysers, with the minimum of operator involvement
  (11) Automatic colorimeters reading out in concentration units but with no automation of chemistry

C. Types of data processing equipment
  (1) Laboratory management computer systems
  (2) Desk-top computer/processors

D. Laboratory situation
  (1) In developed countries with a high level of economy and technology, a good electricity supply, good climatic conditions, etc.
  (2) In developing countries with a low level of economy and technology, electricity supply of variable quality or subject to repeated failure; hot, humid or dusty climate

### Discussion

**Large laboratory users (A1, D1)**

It is now generally accepted that all laboratories in these categories would wish to install as much automation as possible. Until recently this would include a large multi-channel analyser with supporting automation and with all operations linked to a laboratory management computer. Whether the large machine (B5) falls into categories B1, B2, B3 or B4, depends upon personal choice, the choice between B1 and B2 resting entirely with the laboratory; user involvement is required for a decision between B3 or B4, depending upon a preference for operating in screening mode or not. Recently, with the decreasing cost of computers and the increasing efficacy of their application, a number of the newly available high-speed, versatile, single channel kinetic analysers (B7) can now be operated together with considerable effect. This has the advantage of flexibility and of requiring a smaller initial capital outlay. In addition the laboratory is not solely dependent on the reliability of a single large instrument.

**Medium laboratory users (A2, D1)**
The value of the large instruments in these laboratories is marginal. There may be advantages where the circumstances favour an element of screening; a large prestigious machine may serve to attract clients in the private medicine sector, or a large discretionary analyser with a large analytical repertoire may have advantages where there are problems in the availability of suitable workers to cover the same workload by other means. These considerations apart, instruments in categories B6, B7 and B8 are usually appropriate.

Again, it may be difficult to justify the installation of a large management computer (C1), and a system incorporating one or more C2 type processors would be adequate. These machines now have remarkable performance capabilities.

For this size of laboratory, instruments using entirely pre-packaged reagents may be cost-effective. The quality control, and much of the worry, of running the chemistry side of laboratory management is then transferred to the manufacturer. This aspect must have considerable appeal in a situation where the scientific side of laboratory work is more a means to an end rather than an end in itself. Such a situation would exist in a private laboratory run by a physician or in a purely commercial laboratory. There can be definite advantages in this type of operation for all laboratories both in terms of cost effectiveness and overall efficiency, but the advantages may not be clearly apparent. One manufacturer offers a computer program which can be applied to any individual set of circumstances to analyse advantages and disadvantages. (see paper VI).

**Small laboratory users (A3, D1)**
Even laboratories with quite small workloads, if they are in the so-called developed countries, would not be expected to operate without some degree of automation (or mechanisation, depending upon the definition). As workloads decrease, the proportion of control specimens which must be assayed to ensure adequate quality control increases, unless machines (categories B9 and B10) are employed which require the minimum of calibration and control. With decreasing laboratory size, conditions become increasingly favourable for the employment of instruments using pre-packaged reagents, since these can be operated intermittently often without the need for constant re-calibration.

If the capital outlay required for this approach needs to be avoided, a good alternative lies in the employment of limited function automation (e.g. automatic pipettes) by using an automatic colorimeter (category B11) for the final measurement. Mechanically sampling the coloured solution and producing either digitial readout or printout in concentration units eliminates many of the human errors which otherwise can adversely affect the performance of the small laboratory. The employment of automatic electrolyte analysers is advantageous for the same reason.

The small laboratory is at a disadvantage in not having the size of staff to merit the employment of senior individuals capable of maintaining laboratory efficiency at a high level. Therefore it is necessary to utilise easily operated equipment in which a high degree of operating efficiency has been built in by the manufacturer. Modern instruments of this nature are now very reliable but their complex nature means that often even the simplest of breakdowns must be dealt with by the manufacturer's service staff. Unless a good maintenance service is guaranteed, unacceptable delays can occur, while a vital piece of equipment is out of action. Therefore, alternative systems need to be available for such emergencies.

**Users in remote areas or developing countries (D2)**
The growth pattern of clinical chemistry has been such that systems and instruments have been devised in developed countries for use in developed countries, and their suitability for the conditions listed under D2 is almost universally inadequate and often disastrously so. At all levels, high cost is a major consideration, but even when this problem has been overcome for prestige laboratories in the large centres of developing countries, a poor electrical supply, poor climatic conditions, or lack of adequate maintenance can still militate against efficient operation. In such circumstances, however, the difficulties have been overcome by utilising air conditioning, voltage stabilisers and independent electrical generators etc. For the rest of the developing world it must be admitted that suitable instruments are not yet available; though small low-cost robust colorimeters have been purpose designed and should be available soon. In the meantime inadequacies in the currently available equipment must be recognised and allowed for as much as possible.

**Instrumentation for emergency work**
By its nature, emergency work is often of life-saving importance and yet it has to be carried out frequently by harassed and tired staff analysing single specimens or only small runs, often without the quality control procedures which can be applied in normal circumstances. It is therefore important to employ as much mechanisation or automation as possible, using pre-packaged materials if cost effectiveness permits. The large laboratory will usually have a special section for such work utilising equipment in categories B9, B10 and B11, and therefore resembling the small laboratory (A3, D1). The newer types of large analyser have facilities for interposing emergency specimens without interfering with the routine workload.

### Selection of possible instruments

The pace of equipment development has been such that a prospective purchaser is faced at any one time with the prospect of purchasing equipment which might be obsolete when installed. Alternatively he will wait indefinitely for new exciting models promised for the near future; even when these arrive still better ones are usually imminent.

History has shown that a relatively small proportion of the many commercially available instruments are really successful. Unless a laboratory is in a position to be venturesome, e.g. with fallback machines available, or with a remit and finance for extensive evaluation, a laboratory head is advised to purchase well tried instruments from a manufacturer with a good record of reliability, and back-up.

### Collection of detailed information

The laboratory head should prepare a list of equipment which appears to suit his purpose using information obtained from advertisements or exhibitions; as many colleagues as possible with similar problems should be consulted.

Since, because of considerations listed earlier, purchasers must largely make up their own minds when selecting their equipment, the IFCC Expert Panel on Instrumentation has appreciated the inadequacy of much descriptive literature produced by manufacturers, and has guidelines at an advanced stage of preparation, which hopefully will be used by all manufacturers when producing descriptive literature for various groupings of equipment. Advanced drafts of these guidelines will shortly be published in *Clinica Chimica Acta*. Since these have been circulating for comment for several months, company literature based on these draft guidelines may appear quite soon.

